# Development of a ribavirin dosing regimen in transplant recipients with chronic hepatitis E virus infection: a population pharmacokinetic and -dynamic model

**DOI:** 10.1093/jac/dkaf183

**Published:** 2025-06-24

**Authors:** Midas B Mulder, Martijn van Noort, Robert A de Man, Nassim Kamar, Joep de Bruijne, Marjolein Knoester, Hans Blokzijl, Thomas Vanwolleghem, Laurence Roosens, Jacques Izopet, Peggy Gandia, Annemiek A van der Eijk, Herold J Metselaar, Maurice J Ahsman, Tamara J van Steeg, Dennis A Hesselink, Brenda C M de Winter

**Affiliations:** Department of Hospital Pharmacy, Erasmus MC, University Medical Center Rotterdam, Rotterdam, The Netherlands; Erasmus MC Transplant Institute, University Medical Center Rotterdam, Rotterdam, The Netherlands; Erasmus MC Rotterdam Clinical Pharmacometrics Group, University Medical Center Rotterdam, Rotterdam, The Netherlands; LAP&P Consultants BV, Leiden, The Netherlands; Erasmus MC Transplant Institute, University Medical Center Rotterdam, Rotterdam, The Netherlands; Department of Hepatology, Erasmus MC, University Medical Center Rotterdam, Rotterdam, The Netherlands; Department of Nephrology and Organs Transplantation, CHU Rangueil, INSERM UMR 1291, Toulouse Institute for Infectious and Inflammatory Diseases (Infinity), Université Paul Sabatier, Toulouse, France; Department of Gastroenterology and Hepatology, University Medical Center Utrecht, Utrecht, The Netherlands; Department of Clinical Microbiology and Infection Prevention, University of Groningen, University Medical Center Groningen, Groningen, The Netherlands; Department of Gastroenterology and Hepatology, University of Groningen, University Medical Center Groningen, Groningen, The Netherlands; Department of Gastroenterology and Hepatology, University Hospital of Antwerp, Edegem, Belgium; Viral Hepatitis Research Group, Laboratory of Experimental Medicine and Pediatrics, University of Antwerp, Antwerp, Belgium; European Reference Network on Hepatological Diseases (ERN RARE-LIVER), Department of Gastroenterology and Hepatology, University Hospital of Antwerp, Edegem, Belgium; Laboratory for TDM and Toxicology, Antwerp University Hospital, Edegem, Belgium; Department of Virology, CHU Purpan, INSERM U1043, IFR–BMT, University Paul Sabatier, Toulouse, France; Department of Toxicology, CHU Purpan, INSERM U1043, IFR–BMT, University Paul Sabatier, Toulouse, France; Department of Virology, Erasmus MC, University Medical Center Rotterdam, Rotterdam, The Netherlands; Erasmus MC Transplant Institute, University Medical Center Rotterdam, Rotterdam, The Netherlands; Department of Hepatology, Erasmus MC, University Medical Center Rotterdam, Rotterdam, The Netherlands; LAP&P Consultants BV, Leiden, The Netherlands; Quantitative Pharmacology and Pharmacometrics, Merck & Co., Inc., Rahway, NJ, USA; LAP&P Consultants BV, Leiden, The Netherlands; Erasmus MC Transplant Institute, University Medical Center Rotterdam, Rotterdam, The Netherlands; Department of Internal Medicine, Division of Nephrology and Transplantation, Erasmus MC, University Medical Center Rotterdam, Rotterdam, The Netherlands; Department of Hospital Pharmacy, Erasmus MC, University Medical Center Rotterdam, Rotterdam, The Netherlands; Erasmus MC Transplant Institute, University Medical Center Rotterdam, Rotterdam, The Netherlands; Erasmus MC Rotterdam Clinical Pharmacometrics Group, University Medical Center Rotterdam, Rotterdam, The Netherlands

## Abstract

**Objectives:**

The optimal ribavirin dosing regimen for the treatment of chronic hepatitis E virus (HEV) infection in solid organ transplant (SOT) is unknown. We modelled ribavirin plasma concentrations versus virologic response and haemoglobin concentrations.

**Patients and methods:**

Data were collected in a retrospective, multicentre study of adult SOT recipients with chronic HEV infection treated with ribavirin between September 2009 and November 2019. Population pharmacokinetic and pharmacodynamic analyses were conducted using nonlinear mixed-effects modelling. Simulations were performed to select the most suitable RBV dosing regimen considering efficacy and safety.

**Results:**

In total, 107 chronically HEV-infected SOT recipients with 305 ribavirin plasma levels, 592 viral load and 443 haemoglobin concentrations were included. Sustained virologic response was achieved in 68.2% of the subjects. Owing to a low IC_50_, the decline in viral load was independent of ribavirin concentration and dose, whereas haemoglobin decreased with increasing ribavirin concentration and dose. A model-supported ribavirin dose for 180 days of 600 mg/day and kidney function (eGFR) ≥ 60 mL/min/1.73 m^2^, 400 mg/day and eGFR 30–59 mL/min/1.73 m^2^ and 200 mg/day and eGFR ≤30 mL/min/1.73 m^2^ showed good efficacy and low toxicity.

**Conclusions:**

This study constitutes a valuable first step in determining the optimal ribavirin treatment regimen for chronic HEV infections in SOT recipients. Our model suggests a lower dose of ribavirin and longer treatment duration compared to the suggested dosing regimen in the EASL Clinical Practice Guidelines on HEV infection. Implementing our dosing regimen in clinical practice will allow for lower toxicity rates, improved tolerability and equal efficacy in chronically HEV-infected SOT recipients.

**Clinical trial number:**

MEC-2018-1326

## Introduction

Hepatitis E virus (HEV) infection is one of the most common causes of acute viral hepatitis worldwide, and several studies have shown that the number of reported HEV infections has increased over the past decade.^1–4^ In immunocompetent individuals, HEV is normally self-limiting.^5^ However, in solid organ transplant (SOT) recipients, HEV can cause chronic hepatitis and cirrhosis if undiagnosed or left untreated.

The current clinical practice guidelines on HEV of the European Association for the Study of the Liver (EASL) recommend to lower immunosuppressive drug therapy in SOT recipients with a chronic HEV infection.^5^ This results in a sustained virologic response (SVR) in approximately one-third of the SOT recipients.^6^ If this is not possible or unsuccessful, a 3-month course of (off-label) ribavirin (RBV) is recommended.^7–10^ RBV inhibits HEV replication *in vitro.*^11^  *In vivo*, first-line RBV therapy was associated with an SVR in 81.2% of 255 patients.^12^ However, the use of RBV is limited by its side effects: mood disturbances, sleeping disorders, neuropathy and (severe) haemolytic anaemia. The latter is dose-dependent and often necessitates RBV dose reduction or discontinuation.^13–15^

To investigate the population pharmacokinetics and pharmacodynamics of RBV, cases of chronically HEV-infected SOT recipients treated with RBV were collected retrospectively. The associations between RBV plasma concentrations versus HEV virologic response and haemoglobin concentrations were modelled and dosing regimens simulated to optimize RBV treatment in SOT recipients, considering efficacy (viral load) and safety (haemoglobin).

## Patients and methods

### Study design and patients

This was a retrospective, multicentre study in which five hospitals participated. Data were collected from adult SOT recipients diagnosed with a chronic HEV infection, who had been treated with RBV between September 2009 and November 2019. For all patients, demographic and clinical parameters and laboratory results were collected. Data from 92 of these cases had been published previously, but the associations between RBV plasma concentrations and virologic response and haemoglobin concentrations were not analysed using nonlinear mixed-effects modelling, and no simulation-based evaluation of dosing regimens was performed.^16^ The decision and timing to treat HEV with RBV, the starting and maintenance dose of RBV, haemoglobin and viral load plasma concentration measurements were determined by the treating physician. Excluded from the pharmacodynamics analysis were haemoglobin measurements after erythrocyte transfusions, and the viral load was not quantified for five SOT recipients. Viral load measurements showing a relapse of HEV in plasma after one or more preceding negative results were excluded as insufficient data were available to develop a satisfactory relapse model.

### Ethics

A waiver was given for this retrospective study by the Medical Ethics Committee of the Erasmus University Medical Center (MEC-2018-1326).

### Software and modelling techniques

Population pharmacokinetic (PK) and pharmacodynamic (PD) analysis was conducted using nonlinear mixed-effects modelling with NONMEM^®^ (version 7.5.0, ICON, Development Solutions, MD, USA). Pirana (version 2.9.9, Certara, NJ, USA) was used as modelling interface, and results were further analysed and visualized in R (version 3.6.1, R Foundation for statistical computing, Vienna, Austria).^17^ Models were compared using the objective function value. In general, the simplest model that described the data adequately and was suitable for the intended use was preferred. A model was accepted only if its goodness-of-fit and visual predictive check or normalized prediction distribution errors were adequate on visual inspection, its parameter values were deemed to be realistic, and shrinkage values [and relative standard errors (RSEs)] were sufficiently low (shrinkage preferably below 30%, RSE preferably below 50%). A bootstrap analysis was performed on the final models with 1000 data sets. There were no PK or haemoglobin observations below the lower limit of quantification (BLQ); viral load observations reported as BLQ were included using the likelihood estimation method (M3).^18^

### Pharmacokinetic, haemoglobin and viral load analysis

**Figure 1.**
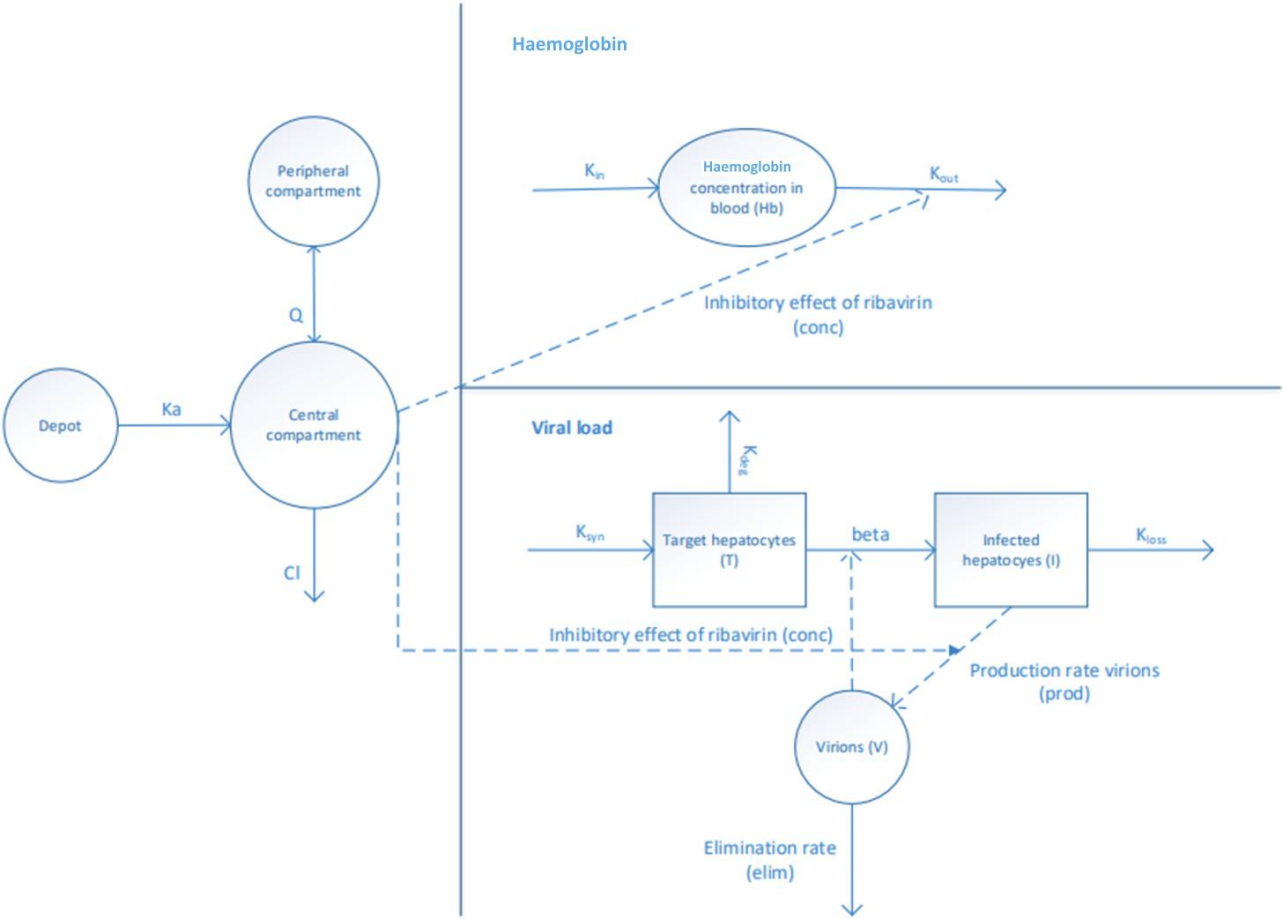
Schematic overview model. Solid arrows indicate mass flow and dashed arrows indicate influence. Ka, absorption constant; Cl, clearance; *Q*, distribution clearance; *k*_in_, production of haemoglobin; *k*_out_, loss of haemoglobin; *K*_syn_, production constant of healthy hepatocytes; *K*_deg_, degradation constant of healthy hepatocytes; *K*_loss_, degradation constant of infected hepatocytes.

A schematic representation of the fully integrated model is provided in Figure 1. Model equations are available in the Supplementary material (available as Supplementary data at *JAC* Online). A two-compartment population PK model with first-order absorption previously developed for RBV in patients with chronic hepatitis C virus infection was used as a starting point.^19^ As RBV plasma samples per patient were limited, the absorption rate constant (*k*_a_), central compartment volume of distribution (*V*_c_), inter-compartmental clearance (*Q*) and peripheral compartment volume of distribution (*V*_p_) were fixed to the estimates published in the starting model.^19^ Due to observed deviations in visual predictive checks, clearance was re-estimated.

Haemoglobin concentrations were modelled using an indirect response model, with a linear inhibitory effect of RBV on the degradation rate (*k*_out_).^19^ The production rate (*k*_in_) was chosen such that baseline haemoglobin concentrations equalled their observed values. In case baseline haemoglobin concentrations were not available (one subject), the typical (median) baseline haemoglobin concentration was used. The parameters *k*_out_ and the slope of the RBV effect were estimated.

The time course of viral load was modelled using a target-cell-limited model with three compartments representing healthy hepatocytes, infected hepatocytes and virions. The inhibitory effect (IC_50_) of RBV on viral replication was described using a sigmoidal Emax model. The viral load was initialized to its observed baseline value, and the fraction of infected hepatocytes was fixed to a low value (rho = 0.001) to prevent unrealistic growth of the liver with clearance of the virus. As the number of hepatocytes was unknown, it was arbitrarily set to one at baseline. To stabilize the model, the elimination rates of healthy hepatocytes (*k*_deg_) and infected hepatocytes (*k*_loss_) were fixed to literature estimates,^20^ and the maximum inhibition of RBV was set to 99.9%. The viral elimination rate (elim) was estimated.

Covariates were selected based on known or theoretical interactions with the pharmacokinetics and pharmacodynamics of RBV. The following covariates were evaluated on CL, *k*_out_ in the haemoglobin model and the elimination rate of virions: sex, weight, age, estimated GFR based on the MDRD, use of mycophenolic acid, liver enzymes (ALT, AST, GGT), bilirubin and albumin. Covariates were tested through a forward inclusion process at significance level *P* = 0.05, followed by a backward elimination with *P* = 0.01. Continuous covariates were centred on the median, and modelled using a power function. Categorical variables were described as multiplicative effects compared to the neutral category (which was either the natural neutral category or the largest category).

Receiver operating characteristic (ROC) curve analyses were performed to determine a cut-off value for the HEV load between SOT recipients with and without SVR. The cut-off value was subsequently used to estimate the minimum treatment duration of RBV achieving viral clearance. The HEV elimination rate at the end of RBV therapy was evaluated for SOT recipients with and without SVR. This might have the potential to distinguish RBV responders from non-responders. Differences in characteristics were described with the Mann–Whitney *U* or Kruskal–Wallis test for quantitative data.

### Simulations

Haemoglobin and viral load Monte Carlo simulations employed the final RBV PK and PD models and established RBV dosing strategies in SOT recipients with chronic HEV infection^10^ (2000 subjects per dosing regimen and sex) in order to assess the optimum dosage and treatment duration for RBV to achieve viral clearance (viral load <100 IU/mL) and prevent severe anaemia (haemoglobin ≤4.9 mmol/L). The remaining covariates, weight and eGFR based on MDRD, were set to their median values, namely 70 (male) and 75 (female) kg and 57 mL/min/1.73 m^2^. Baseline concentrations of haemoglobin per sex and viral load were set to their median values. Different RBV dosing regimens for males and females were assessed.

## Results

### Study population

A total of 107 chronically HEV-infected SOT recipients were included, with 305 RBV plasma concentrations (range 0.1–6.2 mg/L), 443 haemoglobin concentrations and 592 viral loads; 38% (225/592) of the viral load observations were reported as BLQ (Table 1). RBV with a median dose of 600 mg/day (range 100–2400 mg/day) for a median of 3 months (range 1–50 months) resulted in SVR in 68.2% of these patients. A clinician-diagnosed relapse occurred in 8.5% (9/106) of the SOT recipients.

**Table 1.** Characteristics of SOT recipients with chronic HEV infection

	Overall (*n* = 107)
Age, years	56.9 (22–84)
Gender (*n*, %)	
Male	72 (67.3)
Female	35 (32.7)
Body weight, kg	74 (43.5–140)
Kidney function during RBV therapy, ml/min/1.73 m^2^	50 (6–117)
Tacrolimus pre-dose concentration at initiation of RBV therapy, mcg/L	6.2 (2.5–14.3)
Haemoglobin concentration at treatment initiation, mmol/L	8.3 (5.3–11.9)
Viral load at treatment initiation, IU/ml	1 886 058 (527–168 000 000)
Interval between diagnosis of HEV infection and start of RBV, days	128 (1–1507)
Duration RBV therapy, days	90 (21–1333)
Dose RBV, mg	600 (100–2400)
Type of organ transplant (*n*, %)	
Kidney	47 (43.9)
Liver	19 (17.8)
Heart	16 (14.9)
Lung	15 (14)
Kidney and pancreas	4 (3.7)
Kidney and heart	3 (2.8)
Pancreas	1 (0.9)
Lung and liver	1 (0.9)
Lung and heart	1 (0.9)
Immunosuppressive therapy at the start of RBV (*n*, %)	
Tacrolimus	90 (84.9)
Glucocorticoids	76 (71.7)
MPA	62 (58.5)
Everolimus	14 (13.2)
Sirolimus	7 (6.6)
SVR (*n*, %)	
Yes	73 (68.2)
No	30 (28)
Unknown	4 (3.7)

MPA, mycophenolic acid.

Continuous variables are displayed as medians and ranges. Categorical variables as counts and percentages.

### Population pharmacokinetics

Covariates from the starting model (body weight on central and peripheral volume, and sex on peripheral volume) were maintained. Inter-individual variability (IIV) and eGFR were included on clearance. The eGFR effect above an estimated cut-off value of 57 mL/min/1.73 m^2^ was capped at maximum (Table 2). A proportional residual error was estimated. Shrinkage was below 30% for all random effect parameters.

**Table 2.** Estimated population pharmacokinetic and pharmacodynamic parameters for the final model and bootstrap analysis

Model parameter	Description	Population estimate	RSE (%)	Bootstrap of the final model	
				Median	95% CI
Population pharmacokinetic model					
*K*_a_ (h^−1^)	Absorption constant	2.91 (fixed)	—	—	—
CL (L/h)	Clearance	26.4	15	24.3	15.8–36.1
V2 (L)	Volume of central compartment	769 (fixed)	—	—	—
Q1 (L/h)	Distribution clearance to peripheral compartment	104 (fixed)	—	—	—
V3 (L)	Volume of peripheral compartment	3570 (fixed)	—	—	—
eGFR on Cl	Kidney function on clearance	1.32	14	1.22	0.89–1.7
WGT on V2	Weight on volume of central compartment	1.29 (fixed)	—	—	—
WGT on V3	Weight on volume of peripheral compartment	0.725 (fixed)	—	—	—
Sex on V3	Sex on volume of peripheral compartment	0.732 (fixed)	—	—	—
Cut-off value on kidney function (mL/min)	—	57	0.1	57.4	52–180
IIV CL (%CV)	—	50.5	12	47.2	32.9–59.6
Standard deviation proportional error	—	0.377	11	0.375	0.308–0.456
Haemoglobin population model					
*k*_out_ (h^−1^)	Loss of haemoglobin	0.556	26	0.565	0.308–0.911
Slope	The slope of the RBV effect	0.102	11	0.102	0.078–0.129
IIV on *k*_out_ (%CV)	—	463	28.2	443.1	211.7–781.2
IIV on slope (%CV)	—	52.1	28.8	50.6	23.2–92.3
Standard deviation additive error (mmol/L)	—	0.406	8	0.405	0.341–0.473
Viral load population model					
TDEG (h)	Half-life of healthy hepatocytes	6398 (fixed)	—	—	—
Factor	Factor for half-life of infected hepatocytes	100 (fixed)	—	—	—
Elimination rate of virions (h^−1^)	—	0.0123	7	0.0126	0.0090–0.0171
IC_50_ (ng/L)	Half maximal inhibitory concentration	1000 (fixed)	—	—	—
Imax	Maximum inhibition concentration	0.999 (fixed)	—	—	—
Rho	Fraction of infect hepatocytes at baseline	0.001 (fixed)	—	—	—
IIV on elimination rate (%CV)	—	71.7	26.1	70.5	24.6–130
Standard deviation additive error	−	2.01	16.8	1.99	1.33–2.61

CV, coefficient of variation.

For IIV parameters, the estimate is the %CV value, calculated as 100 × √(exp(Ω^2^)−1), where Ω^2^ is the estimated variance, and the RSE is calculated as 100 × SE/(2Ω^2^), where SE is the estimated standard error (SE) for the variance Ω^2^.

### Haemoglobin concentrations

IIV could be estimated on *k*_out_ and the slope of the RBV effect. The residual error model was best described with an additive function (Table 2). Shrinkage was 31% for IIV on *k*_out_ and 36% for IIV on the slope of the RBV effect. No covariate appeared to be relevant on the haemoglobin concentrations.

### Viral load

The IC_50_ parameter of the effect was estimated at a value lower than almost all observed concentrations, which destabilized the model. After a sensitivity analysis (results not shown), this parameter was fixed (1000 ng/L). IIV could be estimated on the elimination rate of virions, with shrinkage below 30%. The residual error model was best described with an additive function on the log scale (Table 2). Visual analysis demonstrated no covariate effect on the elimination of HEV virions.

The ROC curve established a theoretical cut-off point for the viral load of 0.00000372 IU/ml at the end of RBV therapy to indicate whether a SOT recipient will reach SVR (sensitivity 62%, specificity 70%, AUC = 0.677 (95%-CI 0.557–0.797, *P* = 0.005), Figure S1). This value is too low for quantification but illustrates that RBV therapy should be continued when the HEV viral load in blood is negative (<100 IU/ml).

**Figure 2.**
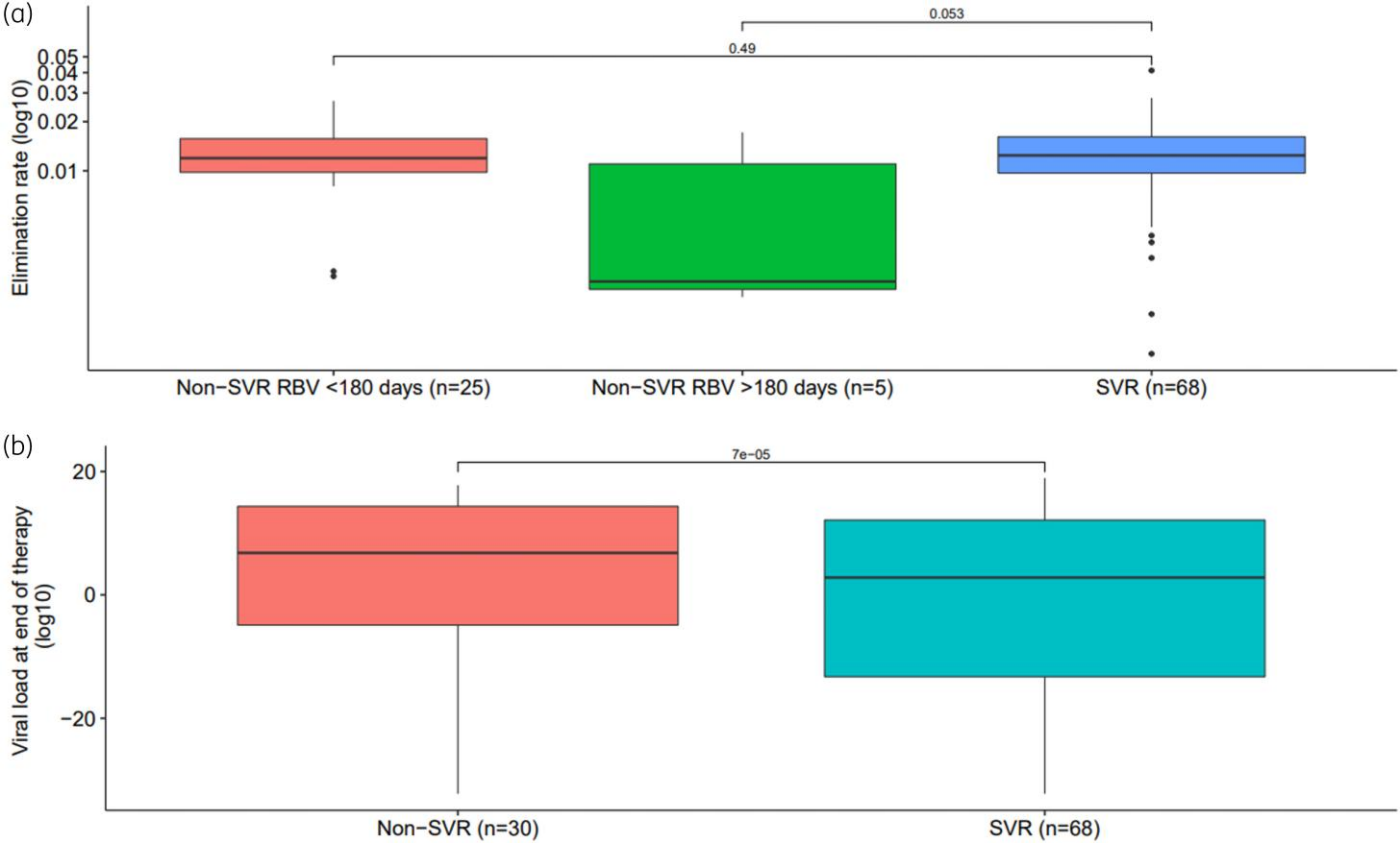
Evaluation of the HEV elimination rate for SOT recipients with and without SVR. HEV elimination rate for SOT recipients with and without SVR (non-SVR) and different treatment durations (< or >180 days).

No difference in the elimination rate between SOT recipients with versus without SVR and a short treatment duration (<180 days) was found (0.012 and 0.011 h^−1^, *P* = 0.49). The elimination rate for SOT recipients with SVR was higher compared to the elimination rate for SOT recipients without SVR who had taken at least 180 days of RBV therapy (0.012 and 0.002 h^−1^, *P* = 0.053) (Figure 2).

### Model diagnostics

Bootstrap analysis was in good agreement with parameter estimates (Table 2). Visual diagnostics showed that RBV concentrations, haemoglobin concentrations and viral load concentrations including BLQ values were predicted by the model with no systematic biases (Figures S2–S7).

### Optimal dosing simulations

**Figure 3.**
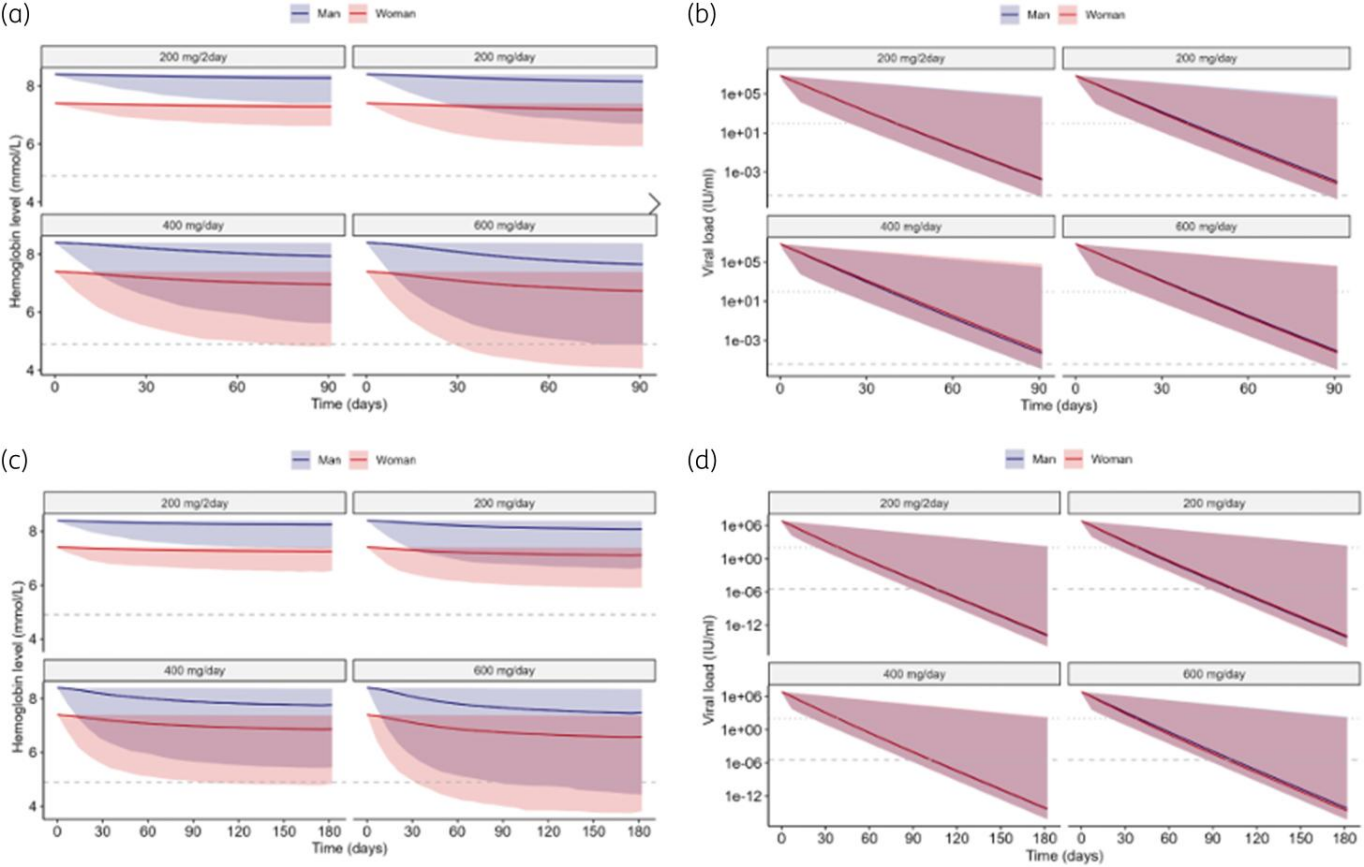
Dose simulation of RBV. (a, c) Simulation of haemoglobin (toxicity) for 90 and 180 days RBV therapy as indicated by the panel header in patients with an estimated glomerular filtration rate of 57 mL/min/1.73 m^2^ . Shaded area represents the 95%-prediction interval. Dashed lines represent the cut-off value of 4.9 mmol/L below which a blood transfusion is recommended. (b, d) Simulation of viral load (efficacy) for 90 and 180 days RBV therapy as indicated by the panel header in patients with an estimated glomerular filtration rate of 57 mL/min/1.73 m^2^ . Shaded area represents the 95%-prediction interval. Dotted lines represent the cut-off value of 100 IU/ml below which the HEV viral load is considered negative. Dashed lines represent the cut-off value established with the ROC curve for the viral load of 0.00000372 IU/ml to indicate whether a SOT recipient will reach SVR. Male and female are overlapping and hard to distinguish.

Figure 3 shows the haemoglobin concentrations and viral loads over time for several dosing regimens. The decline in viral load did not depend on the RBV dose, whereas the decline in haemoglobin was dependent on the RBV dose and the baseline haemoglobin concentration.

The simulated viral load (Figure 3b and d) showed a biphasic profile at the fifth percentile, and a monophasic profile at the 95th percentile. At the upper end of the prediction interval, the rate-limiting elimination rate of the virus drove the viral load decay. A slow viral elimination rate for the virus masked the secondary phase of viral load decay, driven by a decrease in the number of infected cells. For high rates (near the fifth percentile), the RBV-induced reduction in viral production translated into a rapid drop in viral load due to the fast turnover. In a second phase, the slower reduction in the number of infected cells reduced viral load further. As there was no IIV on the elimination rate of infected hepatocytes, all high-rate profiles (including the median) heaped up in the second phase.

After 180 days of RBV therapy, the 95% prediction intervals of the viral load for every RBV dosing strategy were at or below the defined cut-off (<100 IU/ml) where HEV viral load is considered negative (Figure 3d), but 21%–24% of the simulated viral loads were not below the (lower) cut-off point established with the ROC curve for SVR (Figure 3d).

**Figure 4.**
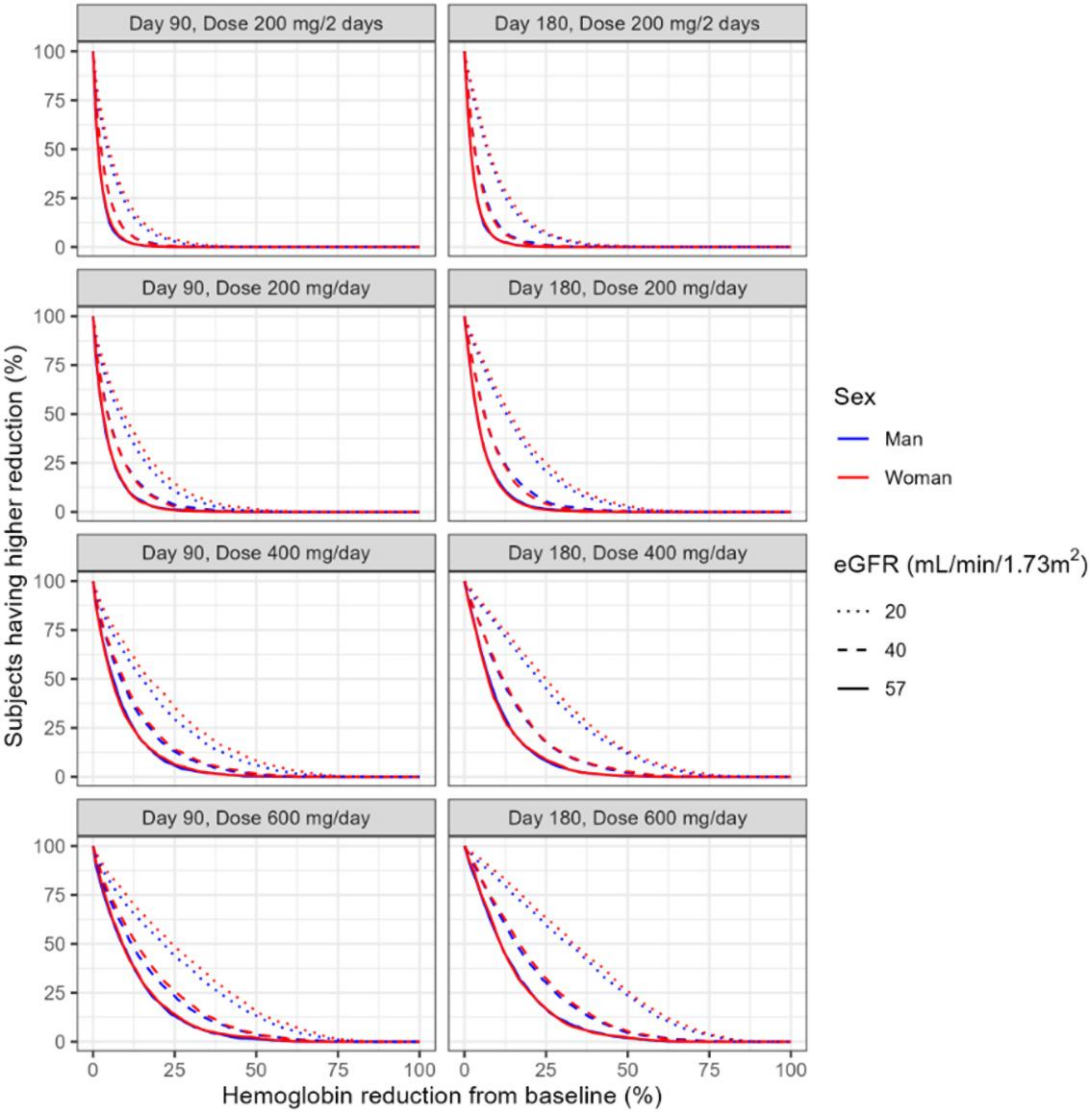
Subjects with more than the indicated reduction in haemoglobin. Percentage of subjects having more than the indicated reduction in haemoglobin, by kidney function (eGFR) and sex, for 90 and 180 days RBV therapy and different dosing regimens as indicated by the panel header; i.e. 25% of the subjects with an eGFR 57 mL/min using 400 mg/day RBV for 90 days have >12.5% haemoglobin reduction from baseline.

Figure 3a and c show large differences in the simulated haemoglobin levels in patients with an estimated glomerular filtration rate of 57 mL/min/1.73 m^2^ with different RBV dosing strategies. For example, for dosing regimens of 200 mg per day or 200 mg every 2 days for 90 or 180 days none of the simulated haemoglobin levels were severely decreased. Figure 4 and Table S1 show the distribution of haemoglobin reduction, by sex, kidney function, treatment duration and dose. The reduction percentages were independent of the haemoglobin baseline, and were similar for men and women.

**Figure 5.**
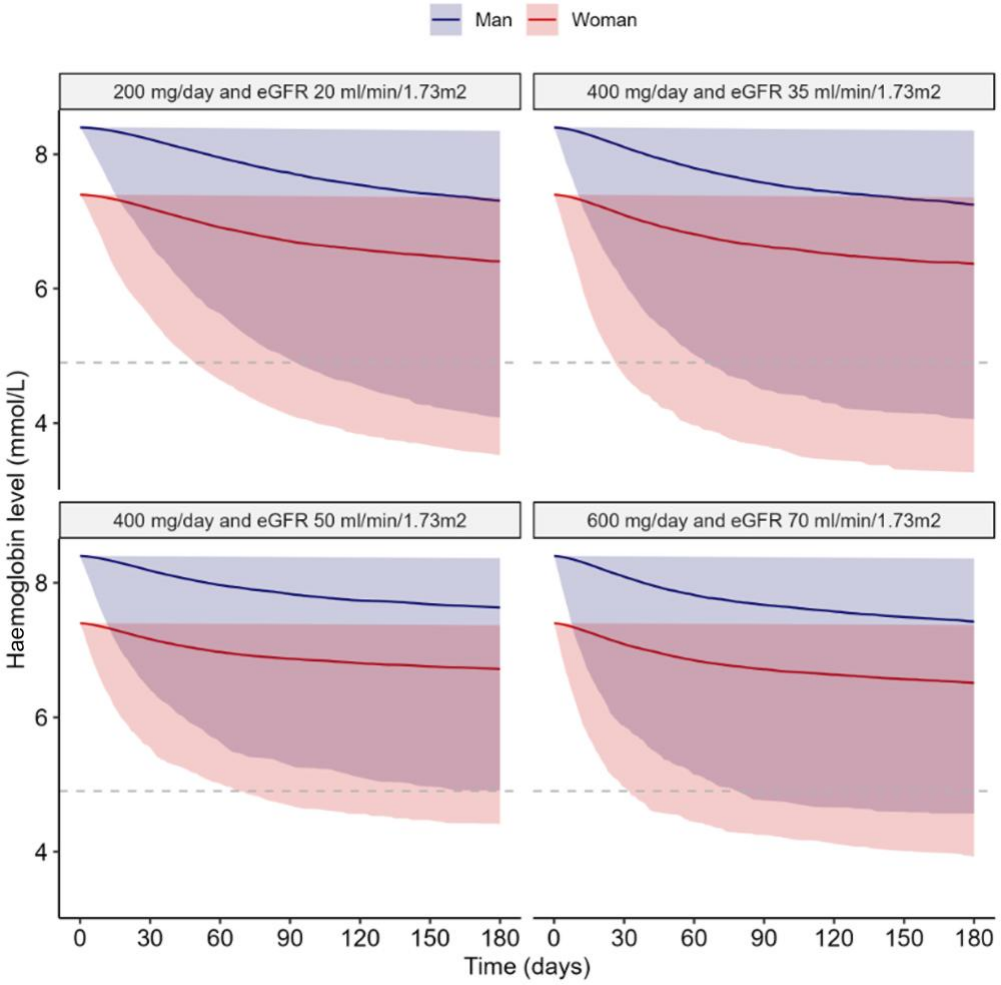
Simulations of haemoglobin for 180 days in SOT recipients with different renal function using 200 mg/day, 400 mg/day and 600 mg/day. Simulation of haemoglobin (toxicity) for 180 days in SOT recipients with different renal function (as indicated by the panel header) using 200 mg/day, 400 mg/day or 600 mg/day RBV. Shaded area represents the 95%-prediction interval. Dashed line represents the cut-off value of 4.9 mmol/L below which a blood transfusion is recommended.

A model-suggested optimized RBV dose of 600 mg/day with a kidney function ≥60 mL/min/1.73 m^2^, 400 mg/day with a kidney function 30–59 mL/min/1.73 m^2^ and 200 mg/day with a kidney function ≤30 mL/min/1.7 3 m^2^ for 180 days showed good efficacy and low risk of anaemia. Figure 5 shows predicted haemoglobin concentrations for 180 days in SOT recipients with different renal functions using 600, 400 and 200 mg/day RBV. The fraction of SOT recipients reaching haemoglobin ≤4.9 mmol/L increased with decreasing renal function. This fraction was higher in women than in men, because of their lower baseline haemoglobin concentrations.

Figures S8 and S9 shows predicted viral load levels and haemoglobin concentrations for 180 days in SOT recipients with different renal functions using 600, 400 and 200 mg/day RBV and starting with a loading dose of 33 mg/kg RBV based on RBV therapy for respiratory syncytial virus infection after lung transplantation.^21^ Including a loading dose did not influence the efficacy (viral clearance) or toxicity (haemoglobin concentrations).

## Discussion

To our knowledge, this is the first population PK/PD model describing the effect of RBV on haemoglobin and viral load in SOT recipients with chronic HEV infection. Currently, RBV therapy for the treatment of chronic HEV infection in SOT recipients is based on case reports and case series, and the optimum RBV dose and treatment duration are unknown.^7,9,10^ Therefore, treatment may benefit from model-based selection of dosing regimens, considering viral load and haemoglobin concentrations. The model predicts viral clearance and acceptable toxicity (haemoglobin >4.9 mmol/L) for this population at 600 mg/day with a kidney function ≥60 mL/min/1.73 m^2^, 400 mg/day with a kidney function 30–59 mL/min/1.73 m^2^ and 200 mg/day with a kidney function ≤30 mL/min/1.73 m^2^. Although RBV doses up to 1200 mg/day are commonly used in clinical practice, in our simulations RBV doses ≥600 mg/day resulted in haematological toxicity without improvement in the viral clearance over the model-suggested dosing regimens. RBV therapy should not be stopped too early as this is illustrated by the theoretical low value for the viral load in the ROC analysis. Furthermore, a lower RBV dose will result in fewer side effects resulting in better treatment compliance.

RBV therapy is associated with dose-dependent anaemia and therefore haemoglobin and eGFR dependent dosing is recommended.^22^ In our model, the haemoglobin concentration decreases with increasing RBV concentrations. RBV-induced anaemia has been studied in patients with chronic hepatitis C virus infection.^23,24^ In one study, bodyweight and haemoglobin concentrations were shown to be relevant covariates in an indirect response model that did not include RBV plasma concentrations.^23^ In another model, plasma and intracellular RBV phosphorylation kinetics were linked to the effect of RBV triphosphate accumulation on red blood cell homeostasis.^24^ Relevant covariates in this model were sex, weight and inosine triphosphatase (ITPA) genotype. In clinical practice intracellular RBV concentrations and ITPA genotypes are not regularly measured. So far, it remains unclear what impact an ITPA variant phenotype has on ribavirin-induced anaemia. Therefore, we developed a model including covariates that will be always available at the start of RBV therapy.

The observed SVR in our cohort (68.2%) was lower compared to another large European retrospective multicentre study with an SVR of 81.2%.^12^ An explanation could be that in our study the immunosuppressive therapy was reduced less strong. The median tacrolimus pre-dose concentration in our study was 6.2 mcg/L. In the other European multicentre study the median tacrolimus pre-dose concentrations were not reported. Another explanation is the use of different combinations of immunosuppressive agents in the studies investigating RBV for chronic HEV infection. The immunosuppressant mycophenolic acid was shown to inhibit HEV replication *in vitro,* but not *in vivo*.^25^ In our cohort, this covariate did not appear to be a significant covariate influencing the elimination of HEV virions. This is possibly due to the high efficacy of RBV.

After the start of RBV therapy, it takes several weeks before steady-state plasma concentrations are reached. Therefore, some centres start with loading doses of RBV for the first couple of days. In patients with HCV, RBV is shown to be a weak inhibitor of HCV viral replication with an IC_50_ between 2.93 and 9.76 mg/L.^26,27^ In contrast, RBV was a strong inhibitor of HEV viral replication in our cohort, and we fixed the IC_50_ at 1000 ng/L after performing a sensitivity analysis evaluating different values for IC_50_. Based on these results, the suggested low IC_50_ of RBV for HEV, and our simulations with a loading dose, we do not advise to include a loading dose since this will not result in faster viral clearance. On the contrary, a loading dose could cause more toxicity (haemoglobin drop) in the first days after the start of RBV therapy. Therefore, the added value of a loading dose in the treatment of chronic HEV is disputable.

Therapeutic drug monitoring (TDM) of ribavirin is hardly performed worldwide whereas this might be of added value as shown in HCV-infected patients by van Tilborg *et al.* and in chronic HEV patients by our research group.^16,28^ Since no correlation between the RBV dose and RBV plasma concentrations at steady state is found and comorbidities, hepatic and/or renal dysfunction, and ITPA gene polymorphism could affect RBV pharmacokinetic, TDM could provide important information on RBV under- or overexposure especially in patients with impaired kidney function (which is the case for most transplant recipients).

Currently, 20%–30% of the chronically infected SOT recipients still do not reach SVR despite RBV therapy. This might be due to insufficient clearance of HEV at the end of RBV therapy as shown by the persistence of HEV RNA in the stool in patients with undetectable HEV RNA in the serum.^5^ We found a 6-fold difference in the HEV elimination rate between SOT recipients with and without SVR, including those with a sufficient treatment duration (>180 days). Further research could investigate whether estimating the HEV elimination rate from two HEV RNA measurements in blood within 6 weeks after the start of RBV could differentiate responders and non-responders to RBV. For non-responders (i.e. patients with a low elimination rate, projected not to reach the clearance threshold within an acceptable time frame), a further reduction in the immunosuppressive therapy might be considered. For responders, RBV should be continued for at least 180 days and thereafter HEV RNA in the stool should test negative twice before considering stopping RBV therapy.

This study has several limitations. First, we did not include SOT recipients experiencing a relapse after ribavirin withdrawal, due to insufficient data. Second, the insensitivity of the viral load model to the value of IC_50_ suggests that the given RBV doses tended to result in maximum suppression of HEV for all or most observations. Third, the use of recombinant erythropoietin and blood transfusions was not included in the model, which could have resulted in an underprediction of the effect of ribavirin on haemoglobin. By correcting for these factors, the model-based dose suggestion would potentially be even lower as proposed now. Finally, as the data were collected retrospectively, we had no control over the dosing regimen, and therefore initial dosing and dose adaptations depended on time varying patient status. This could have affected the results, i.e. an over-estimation of efficacy. SOT recipients who did not achieve viral load reduction would likely be up-titrated during the RBV therapy. A prospective study with controlled dosing of ribavirin and controlled reduction of immunosuppressive therapy before the start of ribavirin would be able to address this.

In conclusion, this study provides a valuable first step in determining the optimal RBV treatment regimen for chronic HEV infections in SOT recipients. Given the model predictions and the data limitations, it seems prudent and feasible to start a non-inferiority, prospective trial evaluating the effect of low dose RBV on HEV clearance in SOT recipients in the near future.

### Study highlights

What is the current knowledge on the topic?

- The optimal ribavirin dosing regimen for the treatment of chronic HEV infection in SOT is unknown.

What question did this study address?

- This study aimed to investigate the population pharmacokinetics and pharmacodynamics of ribavirin in cases of chronically HEV-infected SOT recipients treated with ribavirin. The associations between ribavirin plasma concentrations versus HEV virologic response and haemoglobin concentrations were modelled and dosing regimens simulated to optimize ribavirin treatment in SOT recipients, considering efficacy (viral load) and safety (haemoglobin).

What does this study add to our knowledge?

- A model-supported ribavirin dose for 180 days of 600 mg/day and kidney function (eGFR) ≥ 60 mL/min/1.73 m^2^, 400 mg/day and eGFR 30–59 mL/min/1.73 m^2^ and 200 mg/day and eGFR ≤30 mL/min/1.73 m^2^ showed good efficacy and low toxicity.
- This study constitutes a valuable first step in determining the optimal ribavirin treatment regimen for chronic HEV infections in SOT recipients.

How might this change clinical pharmacology or translational science?

- Our model suggests a lower dose of ribavirin and longer treatment duration compared to the suggested dosing regimen in the EASL Clinical Practice Guidelines on HEV infection.
- Implementing our dosing regimen in clinical practice will allow for lower toxicity rates, improved tolerability, and equal efficacy in chronically HEV-infected SOT recipients.

## Supplementary Material

dkaf183_Supplementary_Data

## Data Availability

Requests for access to the study data can be emailed to the corresponding author.

## Transparency declarations

D.A. Hesselink has received lecture fees and consulting fees from Astellas Pharma, Astra Zeneca, Chiesi Pharma, Medincell, Novartis Pharma, Sangamo Therapeutics and Vifor Pharma. He has received grant support from Astellas Pharma, Bristol-Myers Squibb and Chiesi Pharma (paid to his institution). D.A. Hesselink does not have employment or stock ownership at any of these companies, and neither does he have patents or patent applications. N. Kamar has received speakers fees and participated to advisory boards for Astellas, AstraZeneca, Biotest, CSL Behring, Chiesi, ExeViR, Gilead, Hansa, Merck Sharp and Dohme, Glasgow Smith Kline, Novartis Pharma, Sanofi, Sandoz and Takeda. T. Vanwolleghem is supported by a senior clinical investigator grant from the research foundation Flanders (grant number 18B2821N).

## Supplementary data

Figures S1 to S9 and Table S1 are available as Supplementary data at *JAC* Online.
