# Supplementary material for: Development of a ribavirin dosing regimen in transplant recipients with chronic hepatitis E virus infection: a population pharmacokinetic and -dynamic model
Source: J Antimicrob Chemother. 2025 Jun 24;80(8):2158–68. doi: 10.1093/jac/dkaf183 (PMC12313452; doi:10.1093/jac/dkaf183)

# **Supplementary materials “Development of a ribavirin dosing regimen in solid organ transplant recipients with chronic hepatitis E virus infection based on a population pharmacokinetic and pharmacodynamic model”**

**Supplementary table 1. Percentage of subjects with more than the indicated reduction in hemoglobin, by sex, kidney function, ribavirin dose regimens and treatment durations.**

|  | **Dose** | **Hb reduction 0%** | **Hb reduction 20%** | **Hb reduction 40%** | **Hb reduction 80%** | **Hb reduction 100%** |
| --- | --- | --- | --- | --- | --- | --- |
| **Male and kidney function 20 ml/min/1.73m^2^** | 200mg/2days for 90 days | 100 | 5.45 | 0.25 | 0 | 0 |
|  | 200mg/2days for 180 days | 100 | 11.05 | 0.85 | 0 | 0 |
|  | 200mg/day for 90 days | 100 | 17.5 | 2.1 | 0 | 0 |
|  | 200mg/day for 180 days | 100 | 31 | 6.15 | 0.05 | 0 |
|  | 400mg/day for 90 days | 100 | 38.5 | 12.3 | 0.15 | 0 |
|  | 400mg/day for 180 days | 100 | 55.4 | 21.35 | 0.4 | 0 |
|  | 600mg/day for 90 days | 100 | 52.65 | 23.4 | 0.35 | 0 |
|  | 600mg/day for 180 days | 100 | 67.8 | 37.9 | 1.45 | 0 |
| **Female and kidney function 20 ml/min/1.73m^2^** | 200mg/2days for 90 days | 100 | 7.1 | 0.45 | 0 | 0 |
|  | 200mg/2days for 180 days | 100 | 12.55 | 1.35 | 0 | 0 |
|  | 200mg/day for 90 days | 100 | 21.85 | 3.55 | 0 | 0 |
|  | 200mg/day for 180 days | 100 | 33.85 | 7.65 | 0.05 | 0 |
|  | 400mg/day for 90 days | 100 | 45.4 | 15.45 | 0.05 | 0 |
|  | 400mg/day for 180 days | 100 | 59.45 | 24.3 | 0.3 | 0 |
|  | 600mg/day for 90 days | 100 | 56.7 | 27.45 | 0.95 | 0 |
|  | 600mg/day for 180 days | 100 | 70.9 | 40.65 | 2.1 | 0 |
| **Male and kidney function 40 ml/min/1.73m^2^** | 200mg/2days for 90 days | 100 | 1.5 | 0 | 0 | 0 |
|  | 200mg/2days for 180 days | 100 | 2.45 | 0.2 | 0 | 0 |
|  | 200mg/day for 90 days | 100 | 6.9 | 0.65 | 0 | 0 |
|  | 200mg/day for 180 days | 100 | 10.7 | 1.15 | 0 | 0 |
|  | 400mg/day for 90 days | 100 | 19.45 | 3.75 | 0 | 0 |
|  | 400mg/day for 180 days | 100 | 26.85 | 5.75 | 0 | 0 |
|  | 600mg/day for 90 days | 100 | 31.1 | 8.4 | 0.05 | 0 |
|  | 600mg/day for 180 days | 100 | 39.85 | 11.25 | 0.1 | 0 |
| **Female and kidney function 40 ml/min/1.73m^2^** | 200mg/2days for 90 days | 100 | 1.05 | 0 | 0 | 0 |
|  | 200mg/2days for 180 days | 100 | 1.45 | 0.05 | 0 | 0 |
|  | 200mg/day for 90 days | 100 | 6.25 | 0.25 | 0 | 0 |
|  | 200mg/day for 180 days | 100 | 8.35 | 0.35 | 0 | 0 |
|  | 400mg/day for 90 days | 100 | 21.8 | 4.6 | 0 | 0 |
|  | 400mg/day for 180 days | 100 | 27.75 | 6.1 | 0 | 0 |
|  | 600mg/day for 90 days | 100 | 34.55 | 9.3 | 0.05 | 0 |
|  | 600mg/day for 180 days | 100 | 41.95 | 12.4 | 0.05 | 0 |
| **Male and kidney function ≥57 ml/min/1.73m^2^** | 200mg/2days for 90 days | 100 | 0.35 | 0.05 | 0 | 0 |
|  | 200mg/2days for 180 days | 100 | 0.5 | 0.05 | 0 | 0 |
|  | 200mg/day for 90 days | 100 | 2.2 | 0.2 | 0 | 0 |
|  | 200mg/day for 180 days | 100 | 2.95 | 0.25 | 0 | 0 |
|  | 400mg/day for 90 days | 100 | 10.4 | 1.35 | 0 | 0 |
|  | 400mg/day for 180 days | 100 | 13.85 | 1.45 | 0 | 0 |
|  | 600mg/day for 90 days | 100 | 20.45 | 3.35 | 0 | 0 |
|  | 600mg/day for 180 days | 100 | 25.7 | 4.8 | 0 | 0 |
| **Female and kidney function ≥57 ml/min/1.73m^2^** | 200mg/2days for 90 days | 100 | 0.25 | 0 | 0 | 0 |
|  | 200mg/2days for 180 days | 100 | 0.35 | 0 | 0 | 0 |
|  | 200mg/day for 90 days | 100 | 1.95 | 0.1 | 0 | 0 |
|  | 200mg/day for 180 days | 100 | 2.7 | 0.1 | 0 | 0 |
|  | 400mg/day for 90 days | 100 | 10.95 | 1.2 | 0 | 0 |
|  | 400mg/day for 180 days | 100 | 14.2 | 1.6 | 0 | 0 |
|  | 600mg/day for 90 days | 100 | 20.5 | 3.95 | 0 | 0 |
|  | 600mg/day for 180 days | 100 | 25.05 | 4.45 | 0 | 0 |

For example, 33.5% of the male SOT recipients with an eGFR ≥57 ml/min/1.73m2 using 400 mg/day RBV for 90 days experience >20% hemoglobin reduction from baseline (i.e., baseline hemoglobin of 8 mmol/L will drop below 5.6 mmol/L). **Supplementary figure 1. ROC-curve for HEV load as predictor of effect (SVR) in chronic HEV patients treated with RBV**ROC-curve for RBV plasma concentration as predictor of effect in chronic HEV patients treated with RBV. Cut-off point * = 0.00000372 IU/ml, AUC=0.677 (95%-CI 0.557 to 0.797, p=0.005. At this point, sensitivity was 62% and specificity was 70%.

**Supplementary figure 2. Visual diagnostics of population pharmacokinetic model**

*


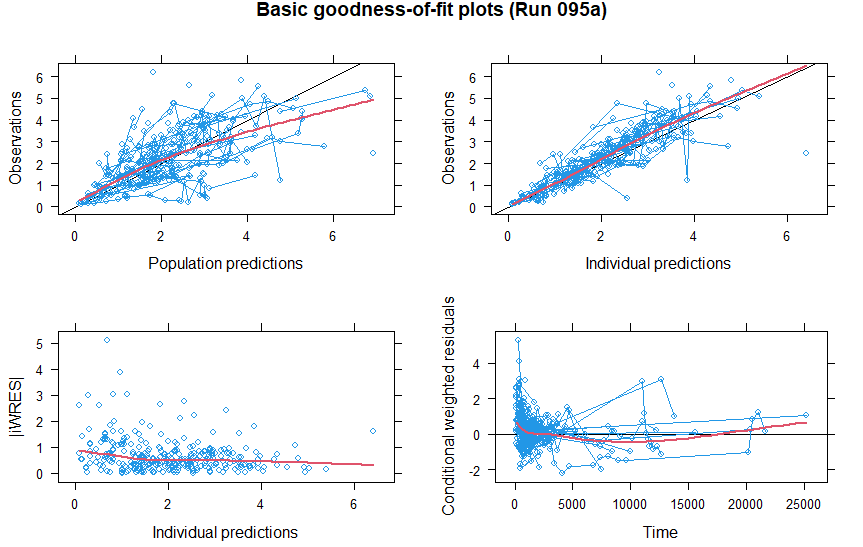


**Supplementary figure 3. Visual predictive check of population pharmacokinetic model**


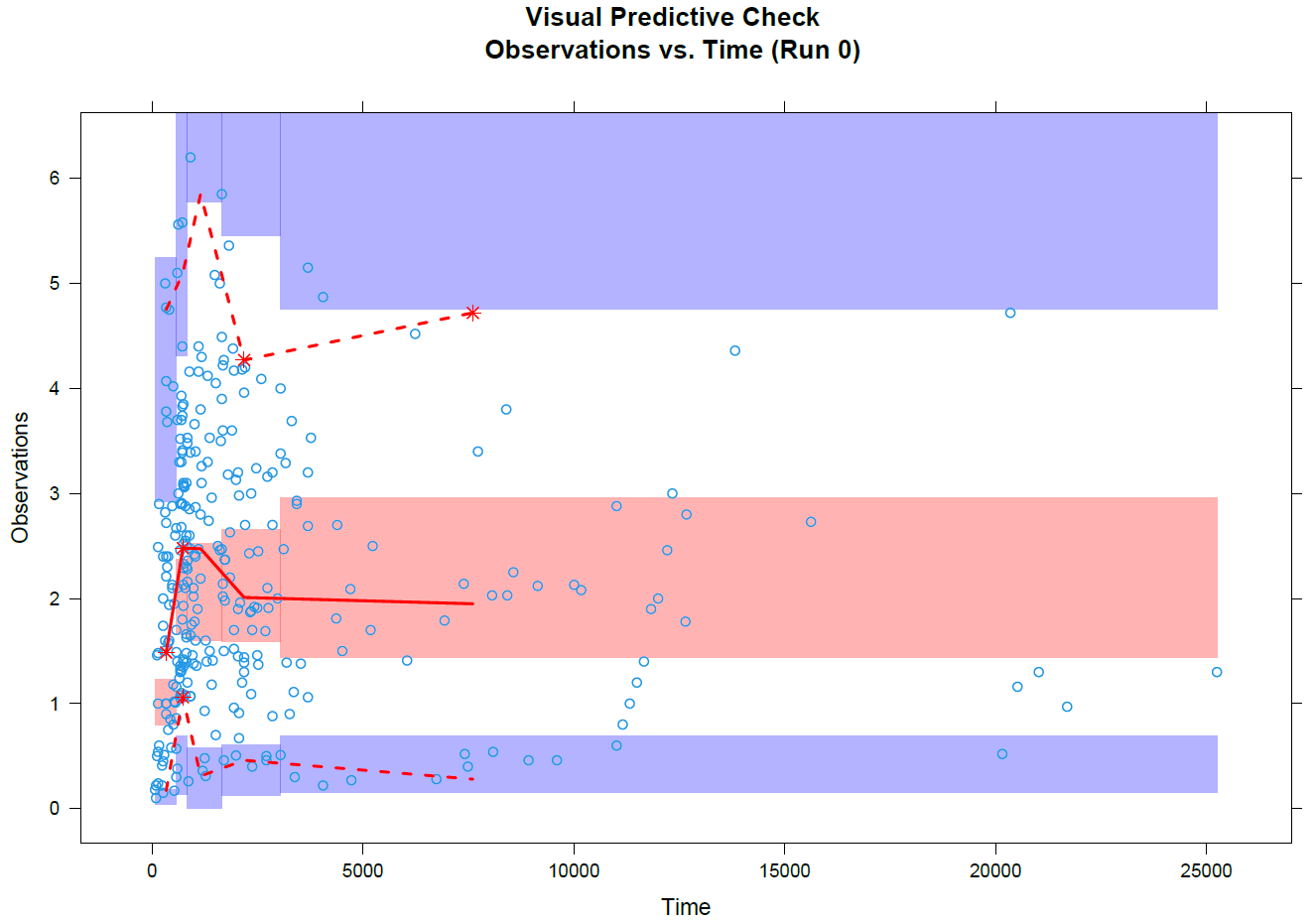


**Supplementary figure 4. Visual diagnostics of the hemoglobin population model**


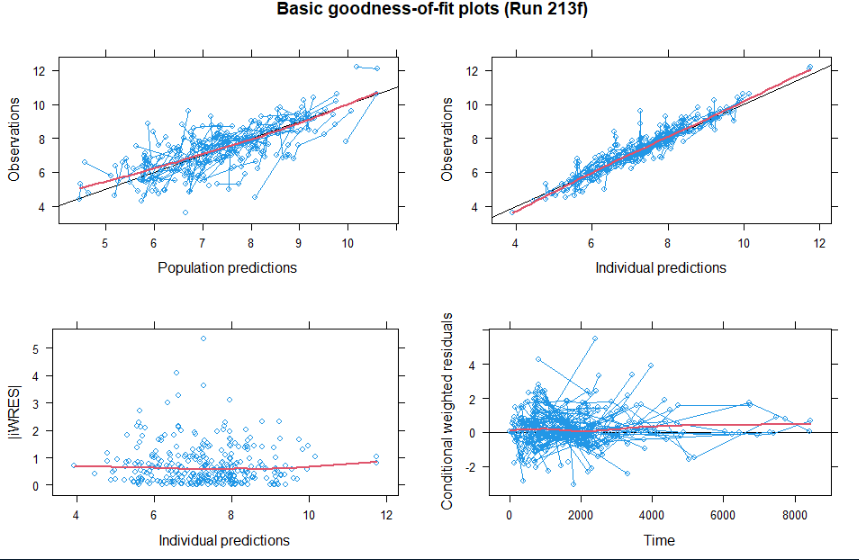


**Supplementary figure 5. Visual predictive check of the hemoglobin population model**


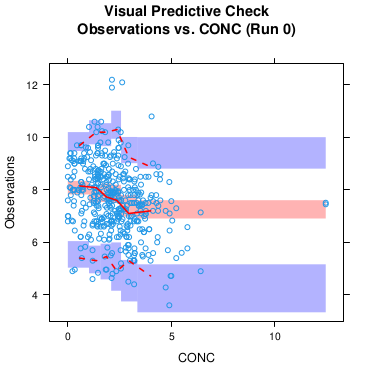


**Supplementary figure 6. Visual diagnostics of the viral load population model**


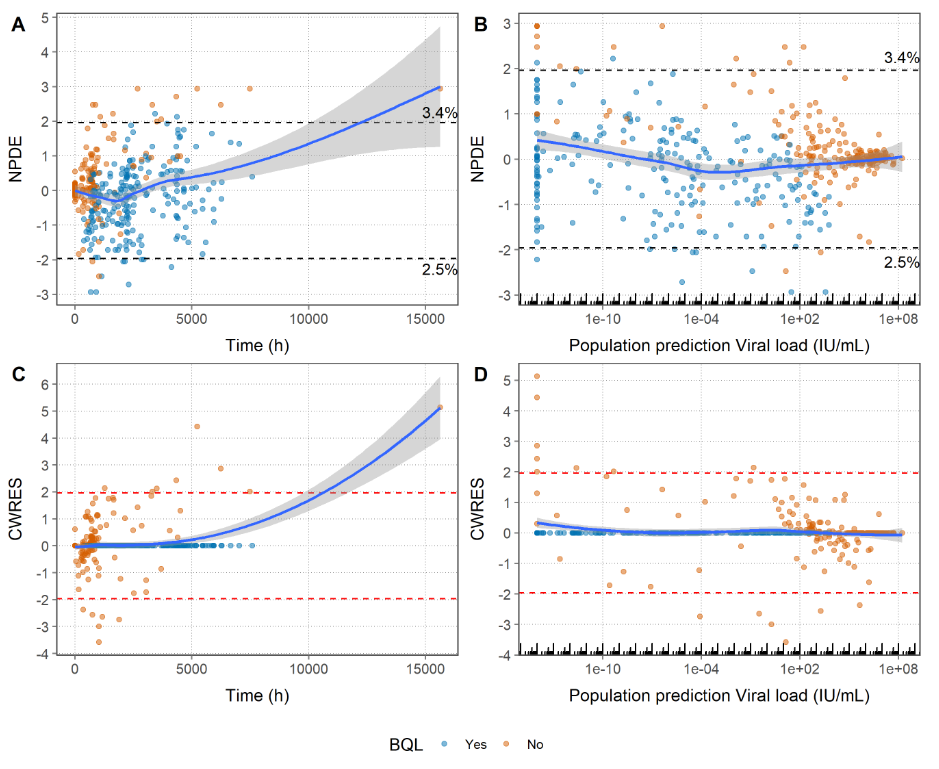


**Supplementary figure 7. Visual predictive check of the viral load population model**


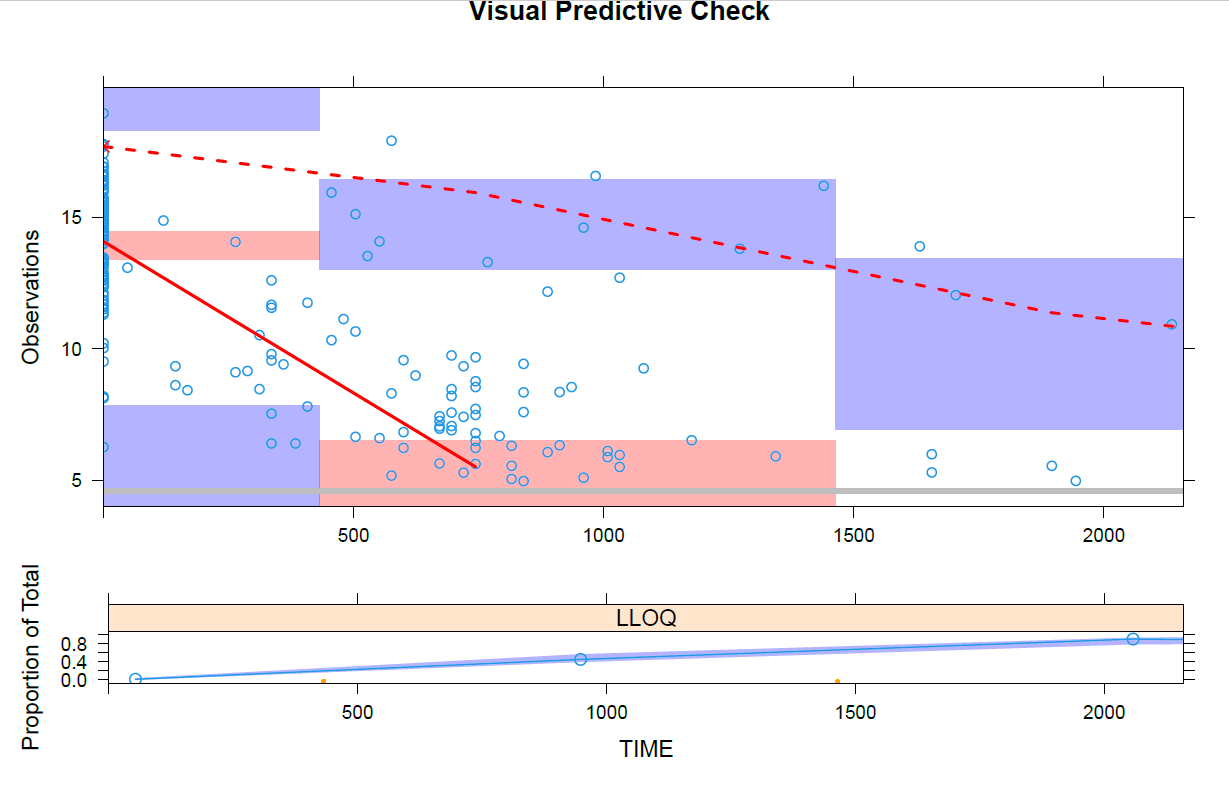


**Supplementary figure 8. Simulations of viral load for 180 days in solid organ transplant recipients with different renal function using 200 mg/day, 400 mg/day and 600 mg/day and a loading dose of 33 mg/kg.**


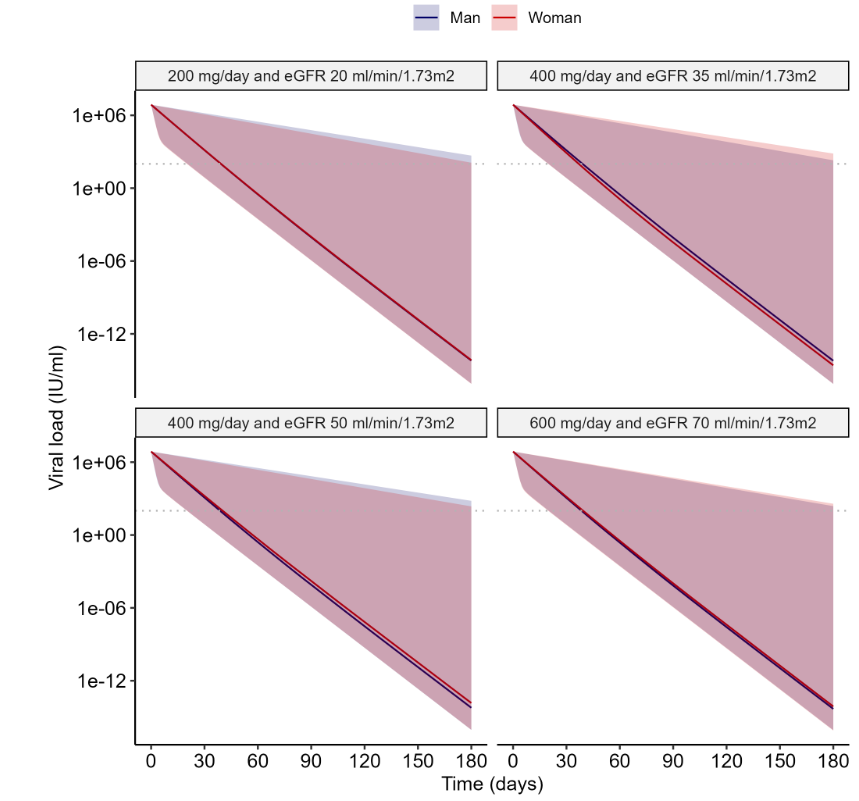


**Supplementary figure 9. Simulations of hemoglobin concentrations for 180 days in solid organ transplant recipients with different renal function using 200 mg/day, 400 mg/day and 600 mg/day and a loading dose of 33 mg/kg.**


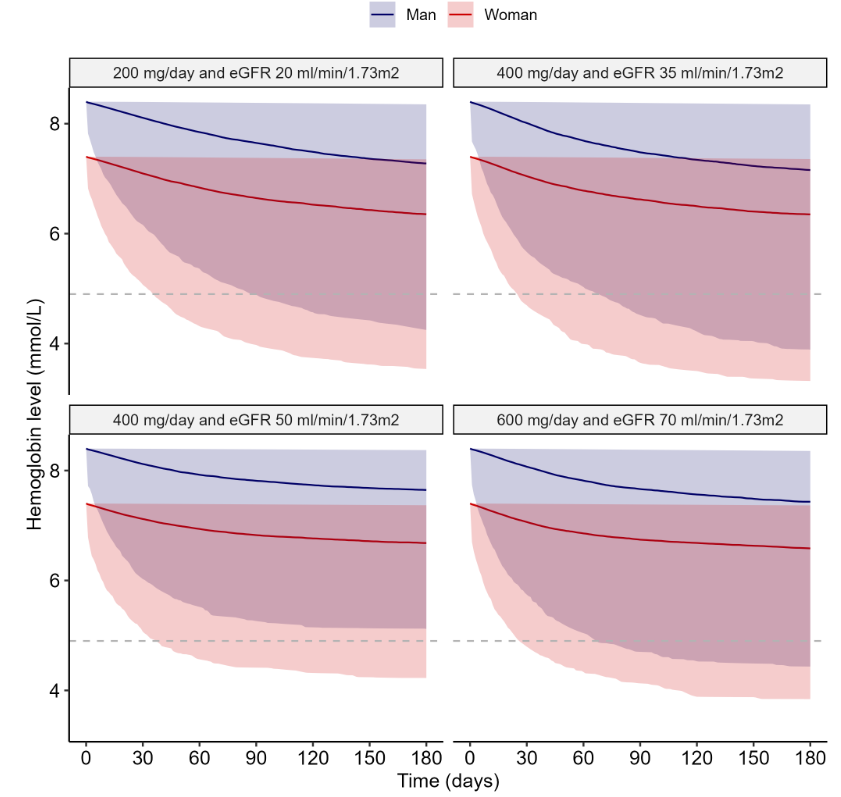

Supplement: dkaf183_Supplementary_Data [file dkaf183_supplementary_data.docx]
